# Mapping of the network connection between sleep quality symptoms, depression, generalized anxiety, and burnout in the general population of Peru and El Salvador

**DOI:** 10.1186/s41155-024-00312-3

**Published:** 2024-07-16

**Authors:** Daniel E. Yupanqui-Lorenzo, Tomás Caycho-Rodríguez, Jonatan Baños-Chaparro, Tania Arauco-Lozada, Luis Palao-Loayza, Marlon Elías Lobos Rivera, Iván Barrios, Julio Torales

**Affiliations:** 1 Universidad Privada del Norte, Trujillo, Perú; 2 Universidad Científica del Sur, Facultad de Psicología, Campus Villa II, Ctra. Panamericana S 19, Villa El Salvador, Lima, Perú; 3 Universidad de Ciencias y Humanidades, Lima, Perú; 4 Universidad de Huelva, Huelva, España; 5 Universidad Tecnológica de El Salvador, San Salvador, El Salvador; 6 Universidad Nacional de Asunción, Facultad de Ciencias Médicas, Cátedra de Psicología Médica, San Lorenzo, Paraguay; 7 Universidad Sudamericana, Facultad de Ciencias de la Salud, Salto del Guairá, Paraguay; 8 Universidad Nacional de Caaguazú, Instituto Regional de Investigación en Salud, Coronel Oviedo, Paraguay; 9 Universidad Nacional de Asunción, Facultad de Ciencias Médicas, Filial Santa Rosa del Aguaray, Cátedra de Bioestadística, Santa Rosa del Aguaray, Paraguay

**Keywords:** Anxiety, Burnout, Sleep quality, Depression, Network analysis

## Abstract

**Background:**

A meta-analysis of randomized controlled trials has suggested a bidirectional relationship between sleep problems and mental health issues. Despite these findings, there is limited conclusive evidence on the relationship between sleep quality, depression, anxiety, and burnout.

**Objective:**

The current study aimed to evaluate the relationships between sleep quality symptoms, anxiety, depression, and burnout in samples of adult individuals from two Latin American countries, Peru and El Salvador, through network analysis and to identify key symptoms that reinforce the correlation and intensify the syndromes.

**Methods:**

A total of 1012 individuals from El Salvador and Peru participated, with an average age of 26.5 years (SD = 9.1). Symptom networks were constructed for both countries based on data from the Jenkins Sleep Scale, Patient Health Questionnaire-2, General Anxiety Disorder-2, and a single burnout item.

**Results:**

The results indicated that Depressed Mood, Difficulty Falling Asleep, and Nervousness were the most central symptoms in a network in the participating countries. The strongest conditional associations were found between symptoms belonging to the same construct, which were similar in both countries. Thus, there is a relationship between Nervousness and Uncontrollable Worry, Anhedonia and Depressed Mood, and Nighttime Awakenings and Difficulty in Staying Asleep. It was observed that burnout is a bridge symptom between both countries and presents stronger conditional associations with Tiredness on Awakening, Depressed Mood, and Uncontrollable Worry. Other bridge symptoms include a Depressed Mood and Nervousness. The network structure did not differ between the participants from Peru and El Salvador.

**Conclusion:**

The networks formed by sleep quality, anxiety, depression, and burnout symptoms play a prominent role in the comorbidity of mental health problems among the general populations of Peru and El Salvador. The symptom-based analytical approach highlights the different diagnostic weights of these symptoms. Treatments or interventions should focus on identifying central and bridge symptoms.

## Introduction

Sleep disorders are a growing public health problem, leading to their consideration as emerging epidemics worldwide, both in developed and developing countries as well as in the general population and clinical settings (Hoyos et al., 2015; Mollayeva et al., 2016; Su et al., 2021). Some studies have suggested that more than 50% of adults in the West experience sleep problems, and approximately 15–20% report chronic sleep problems (Mollayeva et al., 2016). Among these issues, difficulties in falling asleep, maintaining sleep, and non-restorative or poor-quality sleep have been reported (American Psychiatric Association, 2022). During the COVID-19 pandemic, it has been suggested that sleep problems affect approximately 40% of the general population and healthcare workers, with COVID-19 patients reporting the highest prevalence rates of sleep problems (Jahrami et al., 2021). Another study during the pandemic also reported a global prevalence of sleep disturbances of 41%; four out of ten people reported sleep problems during the pandemic (Jahrami et al., 2022). The highest prevalence of sleep problems has been reported in the developed countries. However, it has been suggested that the presence of sleep problems in developing countries may be underestimated and represent an unrecognized public health issue (Su et al., 2021). In this regard, a review and meta-analysis indicated that sleep parameters in low- and middle-income countries are similar to those observed in high-income countries; however, the presence of great variability and bias could lead to errors in the universal estimation of the prevalence of sleep problems (Simonelli et al., 2018). The same study reported that the great variability could not be explained by variables such as region of origin, rurality, sex, age, or methods for assessing sleep.

Sleep quality problems have a negative impact on a range of physical illnesses such as cardiovascular diseases (Khan & Aouad, 2022; Lao et al., 2018), immunological, metabolic, and neuroendocrine alterations (Faraut et al., 2012; Lee et al., 2020), neurocognitive disorders (Lai et al., 2022; Spruyt, 2021), and mental health problems (João et al., 2018; Oh et al., 2019; Scott et al., 2021). Sleep quality and mental health problems are intrinsically related (Baglioni et al., 2016), resulting in significant public health challenges with impacts at the individual and societal levels (Chattu et al., 2018; Hale et al., 2020; Robotham, 2011). It has been suggested that the presence of mental health problems can lead to sleep problems (Stepanski & Rybarczyk, 2006); however, there is also evidence to the contrary, where sleep problems contribute to the onset, recurrence, and maintenance of various mental health problems (Alvaro, Roberts & Harris, 2013; Gregory et al., 2009; Soehner et al., 2013).

Improvements in sleep quality have been reported to lead to improvements in mental health, significantly reducing the presence of anxiety and depressive symptoms (Scott et al., 2021). For example, individuals with insomnia are 10–17 times more likely to exhibit clinically significant levels of anxiety and depression than those without insomnia (Taylor et al., 2005). This was also reported in a meta-analysis of longitudinal studies, where individuals with insomnia had twice the risk of developing depressive disorders than those without insomnia (Baglioni et al., 2011). There is also evidence that sleep quality problems are associated with other mental health issues such as burnout (Chen et al., 2023). Previous studies have indicated that individuals who are dissatisfied with their sleep quality have a higher probability of experiencing emotional exhaustion and depersonalization, two dimensions of burnout (Bagheri Hosseinabadi et al., 2019; Vidotti et al., 2018). Similarly, medical professionals and nurses with high levels of burnout have a higher prevalence of insomnia and poor sleep quality (Membrive-Jiménez et al., 2022; Vela-Bueno et al., 2008). However, other studies have suggested a lack of an association between sleep quality and exhaustion (Mendelsohn et al., 2019; Marek et al., 2019). Based on the existing evidence, the relationship between poor sleep quality and exhaustion is not entirely conclusive. In contrast, a review and meta-analysis reported a significant relationship between exhaustion, depression, exhaustion, and anxiety. Moreover, there was no overlap between the variables, indicating that the three constructs differed (Koutsimani et al., 2019).

A meta-analysis of randomized controlled trials has suggested a bidirectional relationship between sleep problems and mental health issues (Scott et al., 2017). Despite these findings, there is limited conclusive evidence on the relationship between sleep quality, depression, anxiety, and burnout. Previous studies on sleep quality, depression, anxiety, and burnout have the limitation of considering these variables based on the total score on a scale. Therefore, they tend to evaluate the correlation between variables based on an overall score. Obtaining total scores from a scale is based on the assumption that all the items are equivalent. This would ignore the heterogeneity of symptoms, which are represented by the items and have different weights in the occurrence of problems (Tao et al., 2022). Therefore, there is a lack of adequate understanding of the relationship between symptoms (He et al., 2023). In addition, traditional theoretical models, such as the latent variable approach, have a limited capacity to describe bidirectional relationships because they are based on the assumption that variables are relatively independent (Schmittmann et al., 2013). In response to this, the network analysis approach to psychopathology (Borsboom & Cramer, 2013) could clarify specific relationships between symptoms, signs, psychological processes, and personality traits (Fried et al., 2016) and better understand the co-occurrence of sleep quality, depression, anxiety, and burnout. In this study, we focused on the level of symptoms rather than on the psychological processes or traits. For example, unlike methods that combine symptoms, network analysis can reveal how having spasmodic awakenings or being unable to fall asleep can deepen a person's depressive mood. Although the network model has previously been utilized in other scientific disciplines, its incorporation into the behavioral sciences, especially in the analysis of psychopathological variables, was forcefully implemented approximately 10 years ago (Borsboom, 2017; Borsboom & Cramer, 2013; Fonseca-Pedrero, 2018).

Network analysis assumes that symptoms are not the result of a common cause but rather reinforce or inhibit each other (Borsboom, 2017). This can lead to the development of mental disorders and influence daily functioning (Van den Bergh et al., 2021). The network structure allows us to describe how all symptoms interact, which is difficult to achieve in traditional common-cause statistical models (Wang et al., 2021). In network analysis, the network structure is composed of nodes representing symptoms or psychological variables and connecting lines or edges representing statistical relationships between the nodes (Epskamp et al., 2018). The goal of this approach is to identify the central nodes in a network that are more strongly associated with other symptoms (Epskamp et al., 2018) and to identify "bridge symptoms" that could increase the risk of progression from one disorder to another (Cramer et al., 2010). Unlike traditional statistical models, network analysis provides clearer information about the relationships between symptoms (Guineau et al., 2021), visualizes interactions between symptoms (Bringmann & Eronen, 2018), and assesses the importance of different nodes that are interrelated in the network. An important aspect is the treatment of measurement errors in the network analysis model. The presence of measurement errors can lead to reduced power and/or higher rates of false positives. However, the use of cross-sectional network estimation methods, such as the Gaussian graphical model based on the extended Bayesian information criterion (EBIC) employed in the present study and described in the methods section, is robust against the negative impacts of poorly measured variables (Henry & Ye, 2024; Wang et al., 2012). Identifying the presence of central and bridge symptoms would provide information on the symptoms that put individuals at a greater risk of comorbidity (Borsboom & Cramer, 2013). For example, a previous study that applied network analysis indicated that panic and wakefulness problems were the most central symptoms in the network of anxiety, depression, and sleep quality symptoms, and they also had the greatest potential to affect mental health (Wang et al., 2021). Another study reported that the symptom of "being nervous" was strongly related to the symptom "unable to relax," which led to greater sleep impairment (Li et al., 2022). On the other hand, another study based on network analysis indicated that exhaustion, anxiety, and depression are significantly related but also have unique characteristics (Ernst et al., 2021). Specifically, this last study indicated that the anxiety symptoms "Trouble relaxing" and "Being so restless that it's hard to sit still" were related to emotional exhaustion and depersonalization, which are dimensions of burnout.

Despite previous results, it is necessary to conduct studies focused on the association of anxiety, depression, sleep quality, and burnout symptoms in the Latin American context. This is even more important, considering the high prevalence of sleep-related symptoms in Latin America (Bouscoulet et al. 2008). In this regard, a meta-analysis showed that in Latin America, individuals belonging to a lower socioeconomic level (characterized by low-quality education, lower income, and poor quality work) were more likely to suffer from sleep disorders (Etindele Sosso et al., 2023). Recent studies conducted in Peru and El Salvador reported that depression was positively related to insomnia and anxiety (Baños-Chaparro, 2023; Mena & Calderón, 2022), demonstrating a close relationship between symptoms of sleep disorders, depressive disorders, and generalized anxiety disorders in the Latin American region. On the other hand, in Brazil, it has been suggested that burnout and sleep problems significantly explain variations in physical and psychological well-being (Pagnin & De Queiroz, 2015). In this sense, sleep problems and their relationships with other mental health issues could be public health problems in Latin America.

To the best of our knowledge, no studies have explored the relationship between sleep quality, anxiety, depression, and burnout symptoms using network analysis, even less so in the Latin American context. Therefore, the objective of this study was to investigate the network structure of sleep quality, anxiety, depression, and burnout symptoms in samples of adult individuals from two Latin American countries, Peru and El Salvador. Specifically, this study aimed to explore the central and bridging symptoms of this network. Additionally, given the differences in sleep quality, anxiety, depression, and burnout between countries (García-Arroyo & Osca Segovia, 2018; Simonelli et al., 2018; Su et al., 2021; Wright et al., 2022), this study sought to explore the differences in symptom associations between Peru and El Salvador. Understanding how symptoms interact can further characterize the discrepant literature and clarify the complex relationships between sleep quality, anxiety, depression, and burnout. Moreover, this study would allow the identification of central symptoms that affect network maintenance. This new way of understanding the relationships between symptoms allows for the design of novel prevention and intervention strategies and/or aids in the search for etiological mechanisms. Developing interventions for central symptoms would maximize their impact on other symptoms related to central symptoms.

## Methods

### Participants and procedures

The present study analyzed a subset of data that was collected as part of a larger research project called "Study on Psychological Factors Associated with Sleep and Rest," which was approved by the Research Ethics Committee of the University of Sciences and Humanities (approval code: 055-23). The study followed the recommendations of the Declaration of Helsinki (World Medical Association, 1964) and the Code of Ethics of the College of Psychologists of Peru (Colegio de Psicólogos del Perú, 2017). Data from two Spanish-speaking Latin American countries, El Salvador and Peru, were used in this study. The study did not involve invasive or potentially harmful procedures; therefore, the approval of the ethics committee of a single country was sufficient. The study was conducted through an anonymous online survey in Peru and El Salvador during July and August 2023, which was available in Spanish. The inclusion criteria for this study were (a) being of legal age; (b) not having a diagnosis of migraine, sleep disorders, or substance use; and (c) providing informed consent to participate in the study. Information regarding the absence of a diagnosis of migraine, sleep disorders, or substance abuse was self-reported by the participants. All participants were selected using a snowball sampling technique to achieve wider dissemination of the study. The number of participants was calculated using the Monte Carlo simulation method (Constantin et al., 2021), which suggested the participation of at least 710 individuals.

A total of 1012 individuals participated, with an average age of 26.5 years (SD = 9.1). In El Salvador, 561 individuals with an average age of 27.7 years (SD = 8.8) were evaluated. Most of the Salvadoran samples were women (60.2%), single (74.5%), had completed university education (15.7%), or had an incomplete university education (70.6%). In El Salvador, 40.5% had a sleeping schedule, whereas 59.5% did not. Additionally, it was reported that 45.1% of participants had light sleep and 54.9% did not. Finally, Salvadorans indicated an average sleep duration of 6.5 hours (SD = 1.4). Regarding the Peruvian sample, 451 individuals participated, most of whom were women (71.2%), single (85.6%), had incomplete university studies (61.4%), and completed university studies (16.4%). A total of 66.5% indicated that they did not have a sleeping schedule, while 33.5% did. The majority reported not having light sleep (53.7%) and sleeping an average of 6.6 hours (SD = 1.6). Table 1 provides more detailed information on the sociodemographic characteristics of the general sample and each country.

**Table 1:** Demographic characteristics of participants by country and overall

	**El Salvador** (*n*=561)	**Peru** (*n*=461)	**Overall** (*N*=1012)
**Age**	27.7 ± 8.8	24.9 ± 9.3	26.5 ± 9.1
**Sex**			
Male	223 (39.8%)	130 (28.8%)	353 (34.9%)
Female	338 (60.2%)	321 (71.2%)	659 (65.1%)
**Social status**			
Single	418 (74.5%)	386 (85.6%)	804 (79.4%)
Married	87 (15.5%)	33 (7.3%)	120 (11.9%)
Cohabiting	45 (8.0%)	25 (5.5%)	70 (6.9%)
Divorced	9 (1.6%)	3 (0.7%)	12 (1.2%)
Widowed	2 (0.4%)	4 (0.9%)	6 (0.6%)
**Educational level**			
Primary, incomplete	1 (0.2%)	—	1 (0.1%)
Primary, complete	2 (0.4%)	—	2 (0.2%)
Secondary, incomplete	3 (0.5%)	1 (0.2%)	4 (0.4%)
Secondary complete	31 (5.5%)	66 (14.6%)	97 (9.6%)
Technician, incomplete	15 (2.7%)	8 (1.8%)	23 (2.3%)
Technician, complete	25 (4.5%)	25 (5.5%)	50 (4.9%)
University, incomplete	396 (70.6%)	277 (61.4%)	673 (66.5%)
University, complete	88 (15.7%)	74 (16.4%)	162 (16%)
**Sleeping Schedule**			
Yes	227 (40.5%)	151 (33.5%)	378 (37.4%)
No	334 (59.5%)	300 (66.5%)	634 (62.6%)
**Light sleep**			
Yes	253 (45.1%)	209 (46.3%)	462 (45.7%)
No	308 (54.9%)	242 (53.7%)	550 (54.3%)
**Hours of sleep**	6.5 ± 1.4	6.6 ± 1.6	6.5 ± 1.5

### Measures

*The Jenkins Sleep Scale* (JSS-4; Jenkins et al., 1988). The JSS-4 consists of four items that assess the frequency and intensity of different sleep problems over the past 4 weeks, such as difficulty initiating sleep, waking up during the night, waking up during sleep, difficulty maintaining sleep, and waking up exhausted in the morning despite having slept. The items have a six-option Likert-type rating scale ranging from "does not occur to me" = 0 to "occurs to me between 22 and 31 days" = 5. The total score on the JSS-4 ranges from 0 to 20, with higher scores indicating a greater frequency of sleep problems. In this study, the Spanish version of Villarreal-Zegarra et al. (2022) was used, which demonstrated adequate goodness-of-fit indices and α and ω coefficient values for the unidimensional model. In the present study, the JSS-4 showed adequate reliability, using the internal consistency method, both in the Peruvian (ω =.79 [95% CI:.76,.82] ) and Salvadoran (ω =.81 [95% CI:.79,.84] ) samples.

*The Patient Health Questionnaire-2* (PHQ-2; Kroenke et al., 2003). The PHQ-2 consists of the first two items of the PHQ-9 (Kroenke et al., 2001): 1.) Feeling down, depressed, or hopeless, and 2.) Little interest or pleasure in doing things. These items are considered two core criteria for depressive disorders (Staples et al., 2019). The two items have a four-option Likert-type rating scale ranging from 0 (not at all) to 3 (nearly every day), with a total score ranging from 0 to 6. In this study, the Spanish version of Caycho-Rodríguez et al. (2019) was used, which showed adequate reliability. In the present study, the PHQ-2 showed adequate reliability using the internal consistency method for both the Peruvian sample (ω = .76 [95% CI: .70, .81]) and the Salvadoran sample (ω = .78 [95% CI: .73, .82]).

*General Anxiety Disorder 2* (GAD-2; Kroenke et al., 2007). The GAD-2 consists of the first two items of the GAD-7 (Spitzer et al., 2006), which are considered core symptoms of anxiety according to the DSM-V-TR (American Psychiatric Association, 2022): 1.) Feeling nervous, anxious, or on edge, and 2.) Not being able to stop or control worrying. Like PHQ-2, the items of GAD-2 have a four-option Likert-type rating scale ranging from 0 (not at all) to 3 (nearly every day). The total score ranges from 0 to 6. In this study, the Spanish version of Caycho-Rodríguez et al. (2019) was used, which showed adequate reliability. In the present study, the GAD-2 reported good reliability using the internal consistency method for the samples from Peru (ω = .76 [95% CI: .69, .81]) and El Salvador (ω = .85 [95% CI: .81, .88]).

*Single Item for Burnout* (SIB). The SIB was developed ad hoc for this study based on a single item on academic burnout (SIAB; Merino-Soto & Fernández-Arata, 2017). In this sense, content related to education was removed. The final item was as follows: “Please choose the option closest to how you feel. Here, BURNOUT refers to feeling mentally and physically exhausted, as if one were ‘burned out’ from dedicating oneself too much to studies.” The five response options were: 0 = I enjoy my activities. I have no symptoms of burnout; 1 = I am occasionally under stress, and I do not always have as much energy as before, but I do not feel burnout; 2 = I definitely feel burnout, and I have one or more of its symptoms, such as emotional or physical exhaustion; 3 = The symptoms of burnout I am experiencing do not go away. I believe I feel frustrated in my daily activities, and 4 = I feel completely burned out, and I often wonder if I can recover. I am at a point where I may need to make changes or seek assistance.

### Data analysis

Statistical treatment was developed in accordance with the guidelines for psychological network analysis using cross-sectional data (Burger et al., 2023). In particular, the local and global properties of the network, precision of the edges, stability of the centrality index, and comparison of network structures were estimated. The analyses were performed using RStudio software version 4.3.2.

In the preliminary analyses, the sociodemographic variables included in the study were represented by frequencies and percentages for each country and overall. In addition, descriptive statistics, such as the arithmetic mean and standard deviation, were used to summarize the average scores of the psychological variables. For inferential statistics, the networktools package was used to identify potentially redundant node pairs (r > .50) using the goldbricker function, where the Hittner method was used to compare dependent correlations (Hittner et al., 2003; Jones, 2021).

Subsequently, network analysis was conducted in different sections. In this function, the ggmModSelect algorithm, a multivariate estimation of the Gaussian graphical model (GGM) for ordinal data, and the Spearman correlation method were used because they perform better with asymmetric data (Isvoranu and Epskamp, 2023). In particular, ggmModSelect performs an iterative process of 100 random models to select the best GGM based on the extended Bayesian information criterion (EBIC) (Isvoranu and Epskamp, 2023). Non-regularized methods perform adequately in datasets where the number of participants is greater than the number of nodes included in the network, in dense networks (> 40%), and in terms of research questions such as analyzing edges between pairs of nodes and the centrality of an underlying network structure (Burger et al., 2023; Isvoranu and Epskamp, 2023; Williams et al., 2019). The networks were visualized using the qGraph package (Epskamp et al., 2012). The graph design was based on the Fruchterman-Reingold algorithm and the averageLayout function, where nodes are represented as circles and edges are the conditional associations that connect the nodes (Fruchterman and Reingold, 1991). The positive and negative edges are identified in blue and red, respectively. The wider and more saturated the edge, the stronger the conditional association between pairs of nodes. The terms "association" and "conditional association" should not be confused. In networks, an association indicates that two variables are connected in terms of partial correlation. Conditional association refers to the same concept, but with the control of a third ( fourth, fifth, etc.) variable. It is understood that nodes represent variables and edges represent the strength of the conditional association. Thus, the absence of an edge in the model (zero correlation) indicates conditional independence between the two variables after controlling for all the other variables in the network. In psychological research, we are often interested in the strength of relationships in addition to their presence or absence (Epskamp et al., 2022; Isvoranu et al., 2022).

In the second section, the local and global properties of the network are described. Expected influence (EI) and predictability have been reported as measures of local property (Haslbeck and Waldorp, 2018; Isvoranu et al., 2022). The EI was obtained using the centrality function of the qgraph package, which estimates the accumulated importance of the nodes and summarizes the sum of the positive and negative weight values of the edges (Epskamp et al., 2012). This index allows us to identify the nodes that have the greatest centrality in the underlying network structure, implying that they have many strong relationships with other nodes in the network (Isvoranu et al., 2022). In psychopathological terms, it corresponds to the psychological symptom that represents the greatest activation of other symptoms and propagation of information on the network. To estimate predictability, the mgm package and prediction function were used (Haslbeck and Waldorp, 2020). Given the categorical items, normalized precision (nCC) was used to quantify which nodes could be predicted by all their neighboring nodes, representing the practical relevance of the edges connected to a node (Haslbeck and Waldorp, 2018). In terms of global properties, density (D), which represents the strength of the nodes connected in pairs; transitivity (C△), which estimates the global clustering of the nodes; and the average path length (APL), which reflects how information is transmitted between pairs of nodes, were calculated. Finally, the small-world property (S > 1), which analyzes the association between nodes (Isvoranu et al., 2022), was estimated. Global properties were obtained using the smallworldindex function of the qgraph package (Epskamp et al., 2012).

Finally, in the third section, to verify the precision and stability of the estimated networks, a non-parametric bootstrap approach was adopted, where data observations were resampled with replacement to create plausible new data and construct confidence intervals (CI) at a 95% level (Burger et al., 2023). For all analyses, 1000 bootstrap samples were executed using the bootnet package (Isvoranu et al., 2022). To assess the precision and comparison of the edge weight estimates, the bootstrapping method was used. For the stability analysis, a subset bootstrap of case deletion was used, where the stability of the EI index was evaluated after re-estimating the network with fewer cases (Isvoranu et al., 2022). To quantify stability, the correlation stability coefficient (CS) was used, which is the maximum proportion of cases that can be discarded, and the value of the CS should be greater than .25 (Burger et al., 2023). Additionally, a comparison of the network structures of El Salvador and Peru was conducted. The cor function and Spearman’s correlation were used for an initial approximation of the similarity between the networks of each country. Subsequently, with the NCT function incorporated in the NetworkComparisonTest package, a permutation test was applied to compare two independent groups based on 1000 random permutations to test the null hypothesis (van Borkulo et al., 2022). This process was carried out based on the invariance of the network structure based on the maximum statistic (M), which represents the degree to which the network structure as a whole is identical between the groups, and the global strength invariance through the S distance (Si), which is defined as the weighted absolute sum of all the edges of the two networks (Isvoranu et al., 2022). In both tests, the Holm-Bonferroni correction (HB) was used, where a p < .05 indicates statistically significant differences between two underlying networks (van Borkulo et al., 2022).

## Results

### Local and Global Network Properties

The descriptive statistics and network properties are shown in Table 2. Regarding the arithmetic mean, high values for both countries were located in Burnout (_MEl Salvador_ = 2.25, MPeru = 2.37) and Depressed Mood (M_El Salvador_ = 2.08, M_Peru_ = 2.11), and the low values were the same in both countries for Nighttime Awakenings (M_El Salvador_ = .79, M_Peru_ = .88), although they differed in other symptoms such as Difficulty Falling Asleep (M_El Salvador_ = 1.04) and Difficulty in Stay Asleep (M_Peru_ = .88). Similarly, the highest standard deviation in both countries was found in Tiredness on Awakening (SD_El Salvador_ = 1.58, SD_Peru_ = 1.56), although they differed in other symptoms, such as Difficulty in Staying Asleep (SD_El Salvador_ = 1.36) and Difficulty Falling Asleep (SD_Peru_ = 1.33). The lowest values in both countries were for Anhedonia (SD^El Salvador^ = .85, SD_Peru_ = .78), followed by Depressed Mood (SD_El Salvador_ = .93) and Nervousness (SD_Peru_ = .84). No overlapping node pairs were identified in the analysis of node redundancy.

**Table 2:** Descriptive measurements and local and global network properties

**Symptoms**	**El Salvador**								**Peru**							
	**Descriptive**		**Local Property**		**Global Property**				**Descriptive**		**Local Property**		**Global Property**			
	M	SD	EI	P	*D*	C^△^	APL	*S*	M	SD	EI	P	*D*	C^△^	APL	*S*
Difficulty Falling Asleep	1.04	1.32	.94	36.4%	.11	.37	1.47	.87	1.25	1.33	.87	35.6%	.11	.43	1.58	1.11
Difficulty in Stay Asleep	1.07	1.36	.67	43%					.88	1.18	.74	28.9%				
Nighttime Awakenings	.79	1.22	.93	57.4%					.88	1.29	.83	30.2%				
Tiredness on Awakening	1.64	1.58	.84	39.4%					1.70	1.56	.95	22.9%				
Anhedonia	2.06	.85	.78	52.4%					2.08	.78	.82	29.2%				
Depressed Mood	2.08	.93	1.09	49.7%					2.11	.93	1.08	41.2%				
Nervousness	1.97	.94	.95	43.1%					2.06	.84	.87	33%				
Uncontrollable Worry	2.01	.96	.93	42.9%					2.08	.90	.80	34.9%				
Burnout	2.25	1.07	.76	40.7%					2.37	1.01	.69	16.3%				

*M* Mean, *SD* Standard deviation, *EI* Expected influence, *P* Predictability, *D* Density, *C*^*△*^ Transitivity, *APL* Average shortest path length, *S* Small-world index

**Fig. 1:**
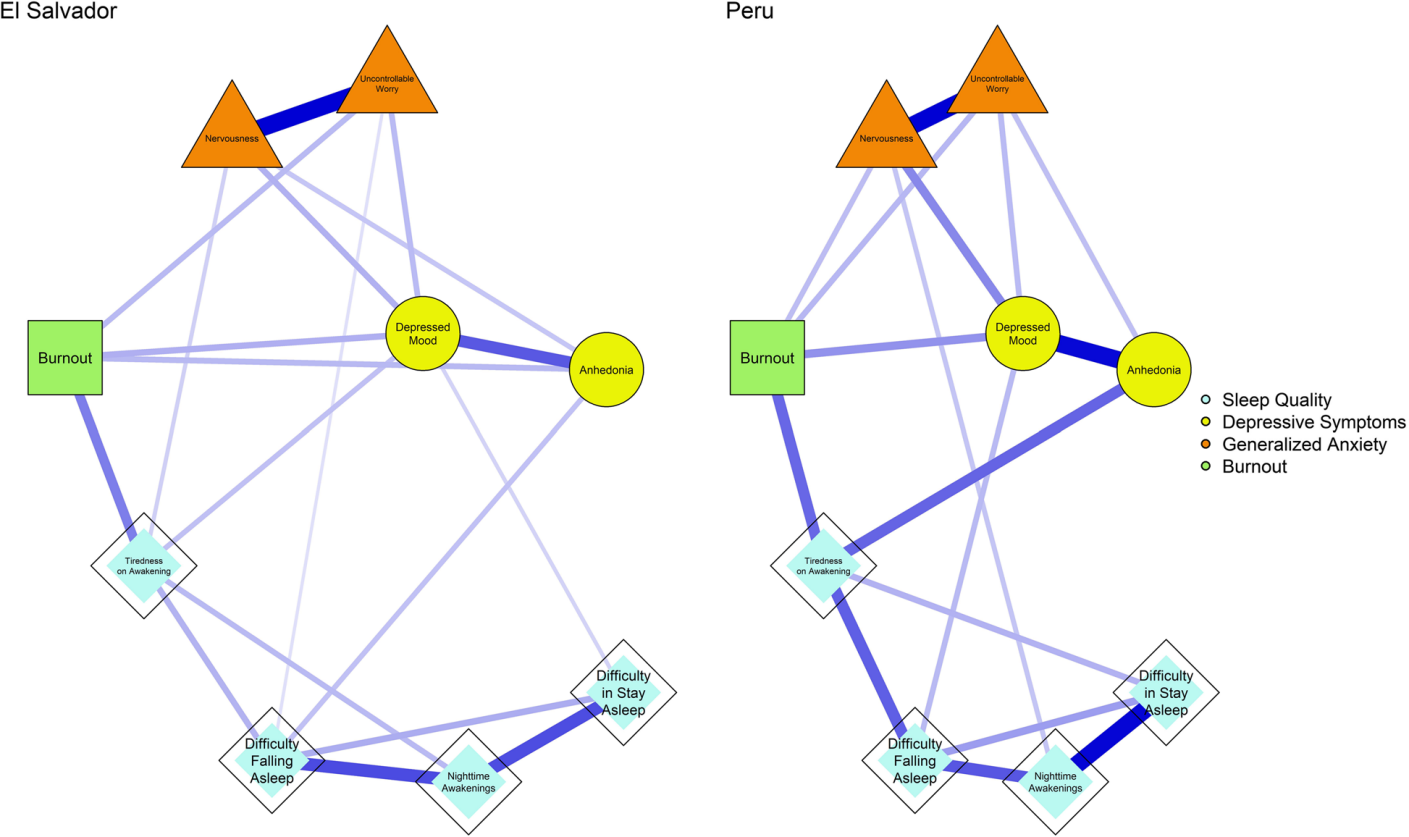
Network structure of sleep quality, depressive symptoms, generalized anxiety, and burnout in adults from El Salvador and Peru

To facilitate a visual comparison of network structures, the nodes were placed in the same location for both countries, as shown in Fig. 1. In both networks, all edges between pairs of nodes were positive; El Salvador presented 19 edges, whereas Peru had 17 edges. Similarly, the strongest conditional associations were found between symptoms belonging to the same construct and were similar in both countries. For example, Nervousness and Uncontrollable Worry (*r*_El Salvador_ = .56, *r*_Peru_ = .45), Nighttime Awakenings, and Difficulty in Stay Asleep (*r*_El Salvador_ = .39, *r*_Peru_ = .44), and between Anhedonia and Depressed Mood (*r*_El Salvador_ = .37, *r*_Peru_ = .43). However, upon further differential exploration, it was found that burnout was relevant in both countries, reporting stronger conditional associations with Tiredness on Awakening (*r*_El Salvador_ = .28, *r*_Peru_ = .26), Depressed Mood (*r*_El Salvador_ = .17, *r*_Peru_ = .19), and Uncontrollable Worry (*r*_El Salvador_ = .15, *r*_Peru_ = .13). Another conditional association that was equal in both countries was Nervousness and Depressed Mood (*r*_El Salvador_ = .17, *r*_Peru_ = .21).

Individually, in El Salvador, Anhedonia was related to burnout (*r*_El Salvador_ = .16) and Difficulty Falling Asleep (*r*_El Salvador_ = .14), as well as Depressed Mood and Tiredness on Awakening (*r*_El Salvador_ = .14). Unlike Peru, where specific associations were found between Anhedonia and Tiredness on Awakening (*r*_Peru_ = .27), Burnout and Nervousness (*r*_Peru_ = .12), and Depressed Mood with Difficulty Falling Asleep (*r*_Peru_ = .12) and Uncontrollable Worry (*r*_Peru_ = .12).

In local network properties, the most central symptoms in EI for both countries were Depressed Mood (IE_El Salvador_ = 1.09, IE_Peru_ = 1.08), Difficulty Falling Asleep (IE_El Salvador_ = .94, IE_Peru_ = .87), and Nervousness (IE_El Salvador_ = .95, IE_Peru_ = .87), although in Peru, Tiredness on Awakening (IE = .95) stood out differently. Meanwhile, in predictability, the only symptom that was the same for El Salvador (49.7%) and Peru (41.2%) was Depressed Mood. Predictability represents the percentage of variance explained by each node given all other nodes in the network, indicating how well a node can be predicted within an underlying network structure. However, the difference was evident in El Salvador with Nighttime Awakenings (57.4%) and Anhedonia (52.4%). In Peru, Difficulty Falling Asleep (35.6%) and Uncontrollable Worry (34.9%) were observed.

Finally, the global metrics were acceptable for a network structure composed of nine nodes in both countries. In particular, the density was similar in each country, given that out of the possible 36 edges, 19 edges (52.8%) were found in El Salvador and 17 edges in Peru (47.2%). The clustering of the nodes was good for El Salvador (C^△^ = .37) and Peru (C^△^ = .43), while the length of the information between pairs of nodes was an average of 1.47 for El Salvador and 1.58 for Peru, which resulted in good transmission in the network dynamics. Additionally, the S index was greater than 1 in the Peru network, but not in El Salvador (S = .87). This suggests that the network structure of Peru presents small-world properties, which represent relatively high levels of C^△^ and small APL lengths between the nodes. The small-world property is defined as having short path lengths for the propagation of information between pairs of nodes and high global clustering among nodes (Barrat & Weigt, 2000).

### Precision of network structure and stability of centrality index

**Fig. 2:**
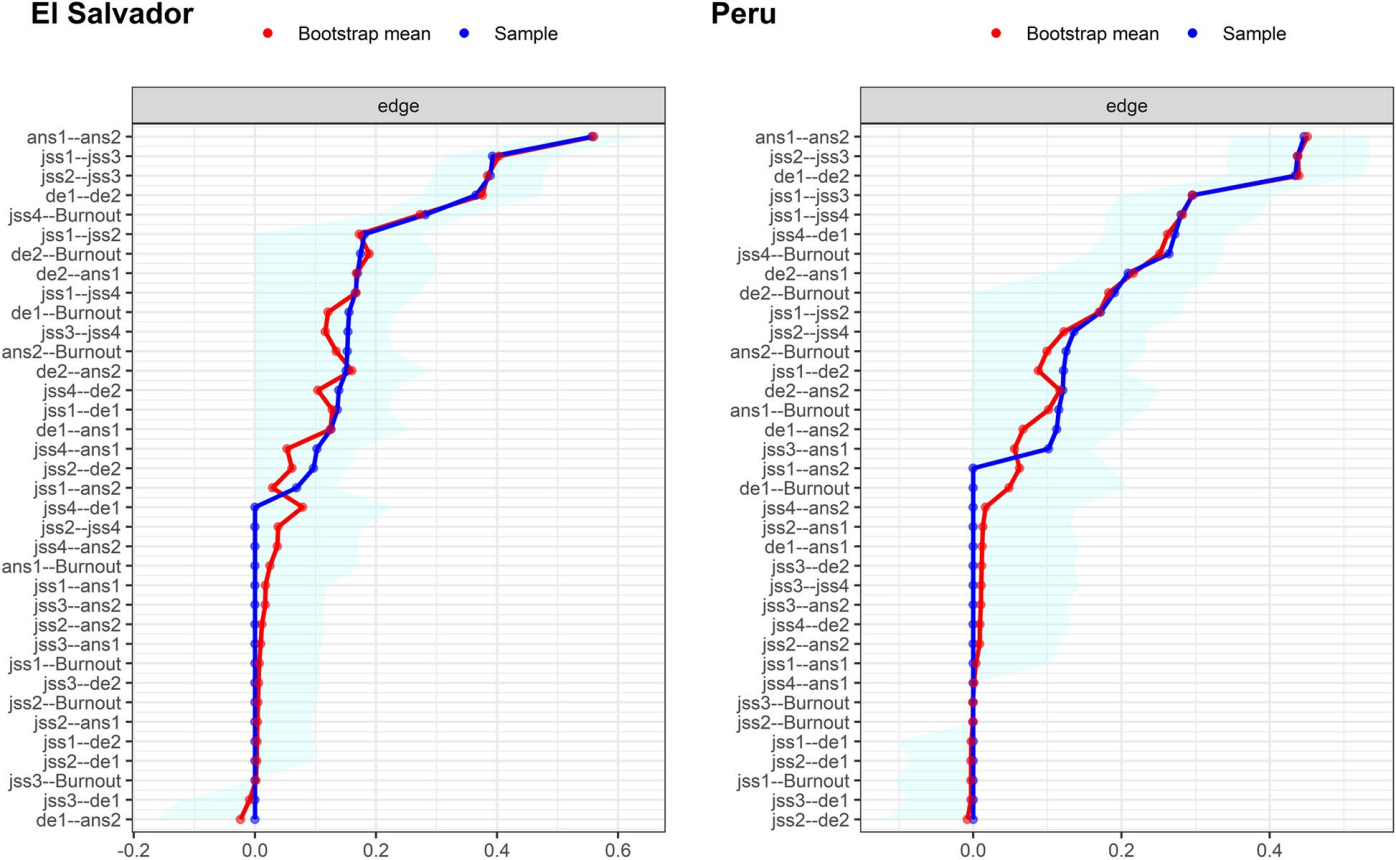
Nonparametric bootstrapping confidence intervals of estimated edges for the network structure

**Fig. 3:**
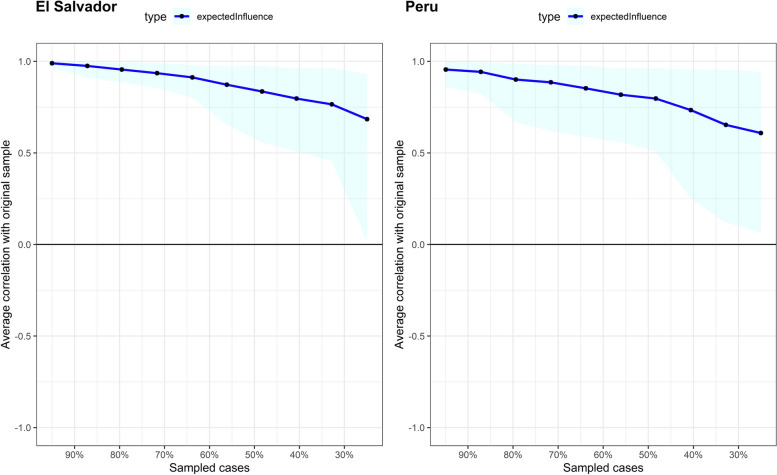
Stability of the expected influence centrality index

In Fig. 2, it is evident that the 95% bootstrap confidence intervals (CI) for the edge weights of the original sample and the resampling average were narrow and maintained stable associations. In Fig. 3, the average correlation of the EI index of the original data and resampling observations with different percentages of cases removed are shown. The stability of the EI was adequate in El Salvador (CS = .439) and Peru (CS = .284), which suggests that they are robust and interpretable.

### Comparative network analysis by countries

The correlation between the network structures of each country was relatively moderate (*r* = .60). The results of the NCT indicated that there was no statistically significant difference in the invariance of network structure (*M* = .16, *p* = .316), although there was a statistically significant difference in the invariance of global strength (*S*_i_ = .27, *p* = .035). In fact, the global strength values for El Salvador (*S*_i_ = 4.05) and Peru (*S*_i_ = 3.78) were notably different, suggesting that there is a stronger global level of connectivity for individual symptoms in El Salvador.

## Discussion

To the best of our knowledge, this is the first study to characterize the network structure of sleep quality and depressive, anxious, and burnout symptoms in adults from two Latin American countries. In the present study, the most central symptoms in both countries were Depressed Mood, Difficulty Falling Asleep, and Nervousness. Thus, these symptoms are important and strongly influence a better understanding of the network structure of sleep quality, depression, anxiety, and burnout symptoms. In both countries, Depressed Mood had the highest predictability.

The finding that Depressed Mood is the most central symptom with the highest predictability has already been reported in previous studies (Bai et al., 2021; Hartung et al., 2019; Liu et al., 2022). This central symptom appears to have the greatest impact and more strongly predicts the appearance of other symptoms. This could be expected because Depressed Mood is one of the most important criteria for the diagnosis of depressive disorder according to the DSM-5, and is characterized by the presence of negative moods, such as feelings of sadness, fear, loneliness, crying, and perceptions of failure (Danhauer et al., 2013). Additionally, Depressed Mood is a common risk factor for the development of comorbid emotional disorders such as depression and anxiety (Naragon-Gainey, 2019). Therefore, it seems that Depressed Mood is one of the most important symptoms that relate to other symptoms. Regarding the symptom of Difficulty Falling Asleep, other studies have also shown that it is one of the most important symptoms of sleep disturbance (Batterham et al., 2012) and is associated with a higher risk of suffering from depression, anxiety, and burnout (Almeida et al., 2011; Narisawa, 2013; Söderström et al., 2012). Regarding burnout, it has been suggested that people who experience burnout report different types of sleep problems, especially difficulties falling asleep and non-restorative sleep (Söderström et al., 2004). Likewise, Nervousness is another symptom that presents greater centrality, suggesting that Nervousness probably affects and/or is affected by modifications to other symptoms (Liu et al., 2022).

Depressed Mood, Difficulty Falling Asleep, and Nervousness, as the most central symptoms, can become important targets within different prevention strategies that can be developed in participating countries. Eliminating or reducing these central symptoms can reduce or prevent activity within the entire network. For example, an action that leads a person to be able to fall asleep would have the potential to improve or prevent their ability to experience pleasure (symptom "Depressed Mood") as well as their ability to not experience Nervousness. In this sense, the network analysis method can generate evidence-based information that can improve interventions based on these central symptoms within the network.

At the general level, the strongest conditional associations were presented between symptoms belonging to the same construct, which were similar in both countries. For example, the strong relationship between Nervousness and Uncontrollable Worry is consistent with the results of previous network studies that evaluated the comorbidity of anxiety symptoms in different populations (Beard et al., 2016; Garabiles et al., 2019; Ren et al., 2021). A strong relationship between these symptoms is expected since both are central symptoms of generalized anxiety, characterized by excessive anxiety and worry about various events, according to the Diagnostic and Statistical Manual of Mental Disorders Text Revision ‑ 5th Edition (DSM-5-TR; American Psychiatric Association, 2022). Similarly, a strong relationship was observed between Anhedonia and Depressed Mood, consistent with previous studies (Savelieva et al., 2021; Su & Si, 2022). It has been suggested that people with symptoms of anhedonia may present a decrease in interest, loss of pleasurable experiences, and depressed mood (Su & Si, 2022). This result is also in agreement with the current diagnostic approach that considers depressed mood and anhedonia as central symptoms for arriving at a diagnosis of clinical depression (American Psychiatric Association, 2022). Finally, a strong relationship was observed between the symptoms of Nighttime Awakenings and Difficulty in Stay Asleep. This has been observed in a previous study in which people with difficulty falling or staying asleep were more likely to have more nighttime awakenings (Biggs et al., 2020). This relationship may be associated with the presence of significant distress or impairment in daytime function, and may occur despite adequate sleep conditions (Patel et al., 2022).

On the other hand, a more thorough differential analysis reported that burnout is a bridge symptom that is relevant for both countries and presents stronger conditional associations with Tiredness on Awakening, Depressed Mood, and Uncontrollable Worry. Additionally, two other bridge symptoms in the current network, which have an equal conditional association in both countries, are the depressive symptom "Depressed Mood" and the anxiety symptom "Nervousness." These findings would indicate that when burnout is present, its treatment could reduce Tiredness on Awakening, Depressed Mood, and Uncontrollable Worry, and vice versa. This has been observed in previous studies (Koutsimani et al., 2019; Söderström et al., 2004). Similarly, treating "depressed mood" may reduce the risk of experiencing nervousness, and when anxiety is present, treating nervousness can reduce the risk of depression. Previous studies on the symptom network of anxiety and depression have also reported that "Depressed Mood" and "Nervousness" are bridge symptoms (Garabiles et al., 2019; Park & Kim, 2020). Although the symptoms of depression and anxiety are differentiated within the network, the interconnection between depressed mood and nervousness may be a transdiagnostic link between them (Park & Kim, 2020).

Finally, the results indicated no statistically significant difference in the invariance of the network structure between Peru and El Salvador. This is important and contrary to the idea of limited replicability of networks in different samples (Forbes et al., 2017), and indicates that the network structures are similar in Peru and El Salvador, which is important for exploring how sleep quality, anxiety, depression, and burnout symptoms manifest in different countries. This finding is important because, despite the impact of different cultural, social, and governmental systems of the countries on the development of different psychological symptoms (Caycho-Rodríguez et al., 2024), the networks did not vary, and the matrices were similar in this study. This would allow for a comparison of the symptom networks of sleep quality, anxiety, depression, and burnout between countries. Having information on the differences or similarities in the symptom networks of sleep quality, anxiety, depression, and burnout between Peruvians and Salvadorans can help develop communication strategies to disseminate preventive messages about sleep quality, anxiety, depression, and burnout problems in these two countries. The evidence that the symptom networks do not vary allows us to know that the possible differences in the relationships between the symptoms are due to the real differences between Peru and El Salvador and are not caused by measurement errors associated with the presence of response biases or differences in understanding each of the symptoms. Despite the invariance in the symptom networks, the results suggest that the connectivity between symptoms is stronger in El Salvador.

In a more connected network, such as that in El Salvador, the activation of one symptom quickly spreads to other symptoms, leading to the emergence of additional symptoms. As a result, Salvadoran participants might more easily suffer from issues such as poor sleep quality, anxiety, depression, and exhaustion. In such circumstances, addressing these mental health problems is more challenging than for participants from other countries. This could be related to the increasing problems of mental illness and the subsequent consequences of these conditions that have been observed in El Salvador in recent years (Molina, López, & Burgos, 2020). Here, it is important to consider the socioeconomic determinants associated with different problems that have affected the country, such as inequality, migration, economic instability, and other determinants related to mental suffering (Molina, López & Burgos, 2020).

The strengths of the present study include the large number of participants in the sample and the use of the network approach to adequately visualize the relationships between sleep quality, depression, anxiety, and burnout symptoms in adults from the two Latin American countries. However, these findings should be interpreted based on some limitations. First, non-probabilistic sampling was used, which would limit the generalization of the findings to the general population of both participating countries. Second, the sample in Peru and El Salvador was predominantly female, unmarried, and university-educated, which could mean that the participants did not fully represent the general population of El Salvador and Peru. In this context, the findings may be more applicable to people with these characteristics in these countries. Therefore, it is recommended to conduct studies with more balanced samples in terms of sex, marital status, educational level, and other characteristics to confirm or refute the findings. Third, the study sample was derived from the general population. While this allows for the analysis of sleep quality, anxiety, depression, and burnout in a dimensional rather than categorical manner, it also limits the generalization of the findings to clinical populations. Future studies should analyze the networks of sleep quality, anxiety, depression, and burnout in individuals with clinically depressed, anxious, or diagnosed sleep disorders. Fourth, the findings are exploratory, similar to most existing network analyses, as they did not test preconceived hypotheses. This limits the extent to which the impact of culture on the relationship between sleep quality, anxiety, depression, and burnout symptoms can be theorized. Fifth, cross-sectional data were used, which limited the detection of directionality in the networks of sleep quality, depression, anxiety, and burnout symptoms. Future studies should evaluate the temporal dynamics among sleep quality, depression, anxiety, and burnout symptoms by incorporating the time variable into the design. In this sense, longitudinal data should be included to estimate the directed networks. However, the use of a cross-sectional sample does not allow for statements about the theoretical development of the network approach or whether the network approach is better than latent disease models. In this study, only statements about the outcome of network statistical analyses were made, not about the network model theory. This is because cross-sectional network models do not allow the recovery of theoretically proposed processes (Fried, 2020; Haslbeck et al., 2022). Previous studies have criticized the reliability of cross-sectional networks (Forbes et al., 2017; Steinley et al., 2017); however, other studies have shown evidence to the contrary, offering procedures to increase the reliability of cross-sectional results (Borsboom et al., 2017; Epskamp et al., 2018), which can be taken into account in future studies.

Sixth, the scales evaluated a limited subset of sleep quality, depression, anxiety, and burnout symptoms. Future studies could use more comprehensive measures with more symptoms for a better understanding of these issues. Seventh, cross-cultural studies have been suggested to generate specific biases, such as instrument bias generated by different levels of familiarity with items and response modes (He & van de Vijver, 2012). Efforts were made to minimize the presence of this type of bias by using the adapted and validated linguistic versions of the JSS-4, PHQ-2, GAD-2, and SIB. Eighth, self-report measures were used to assess sleep quality, anxiety, depression, and burnout, which could generate social desirability or memory bias.

## Implications

Despite the study limitations, the results demonstrated that the use of network analysis has significant theoretical implications for understanding the relationship between symptoms of anxiety, depression, sleep quality, and exhaustion. Various studies have confirmed the relationship between anxiety, depression, sleep quality, and exhaustion (e.g., Haghighi & Gerber, 2019; Koutsimani et al., 2019; Tang et al., 2023). However, as mentioned earlier, these studies rely on traditional conceptualizations of mental health issues, where symptoms reflect the underlying variables. According to this view, the co-occurrence or clustering of symptoms is the product of a common underlying cause (Schmittmann et al., 2013). For instance, under this traditional model, it is hypothesized that depression generates a sad mood or anhedonia, just as a virus causes fever and headaches (Beard et al., 2016). This perspective also supports the use of global scores to describe the severity of a mental health problem, as symptoms are considered to be indicators of the same underlying condition, and therefore, their scores can be summed to produce a total score (Fried & Nesse, 2015). This could mask the presence of significant differences between specific symptoms since the impact of one symptom on others occurs differentially (Fried & Nesse, 2014).

Contrary to traditional models, network analysis suggests that the coexistence of symptoms of anxiety, depression, sleep quality, and exhaustion is due to the presence of direct relationships between symptoms, and not a common cause. Thus, the relationships between different symptoms constitute a substance of mental problems (Borsboom & Cramer, 2013). Theoretically, symptoms of anxiety, depression, sleep quality, and exhaustion directly influence each other and have their own genetic, neural, and psychological bases (Beard et al., 2016).

Practically, results based on network analysis provide new insights into the functional role and importance of certain specific symptoms in the maintenance of diseases, such as the centrality of symptoms within a network (Guo et al., 2023). Detecting communities of central symptoms (depressed mood, difficulty falling asleep, and nervousness) and bridge symptoms (burnout, depressed mood, and nervousness) allows us to understand the symptoms that are most closely related to each other and can be considered potentially relevant intervention targets that could maximize the reduction of sleep problems, anxiety, and depression. For example, the activation of symptoms of a depressed mood can rapidly propagate to other symptoms, causing more symptoms of depression, poor sleep quality, anxiety, and exhaustion over time. However, further research is needed to test the extent to which interventions targeting these symptoms are effective in altering the network structures of sleep quality, anxiety, depression, and exhaustion. It is important to consider that central symptoms are the most challenging to intervene in because of their high degree of connectivity within the network. This is because the activation of any other connected symptom can reactivate and maintain the central symptoms. Therefore, when planning interventions, mental health professionals should focus on central symptoms and identify and address the clusters of symptoms within which they are integrated.

## Conclusion

In conclusion, Depressed Mood, Difficulty Falling Asleep, and Nervousness were the most central symptoms in a network in participating countries. The strongest conditional associations were presented between symptoms belonging to the same construct, which were similar in both countries. Thus, there is a relationship between Nervousness and Uncontrollable Worry, Anhedonia, and Depressed Mood, and Nighttime Awakenings and Difficulty in Stay Asleep. It was observed that burnout is a bridge symptom between both countries and presented stronger conditional associations with Tiredness on Awakening, Depressed Mood, and Uncontrollable Worry. Other bridge symptoms are Depressed Mood and Nervousness. The network structure did not differ between participants from Peru and El Salvador. However, the connectivity between the symptoms was stronger in El Salvador.

## Data Availability

The data presented in this study are available on request from the corresponding author.

## Abbreviations

EBIC: Bayesian information criterion
EI: Expected influence
nCC: normalized precision
D: Density
C△: Transitivity
APL: average path length
CI: Confidence intervals
CS: Stability coefficient
M: Maximum statistic
HB: Holm-Bonferroni correction
GGM: Gaussian graphical model
